# Supplementary material for: Parent-of-Origin inference for biobanks
Source: Nat Commun. 2022 Nov 5;13:6668. doi: 10.1038/s41467-022-34383-6 (PMC9637181; doi:10.1038/s41467-022-34383-6)
Supplement: Supplementary file 1 — Supplementary Information [file 41467_2022_34383_MOESM1_ESM.pdf]

Supplementary information

Supplementary figures

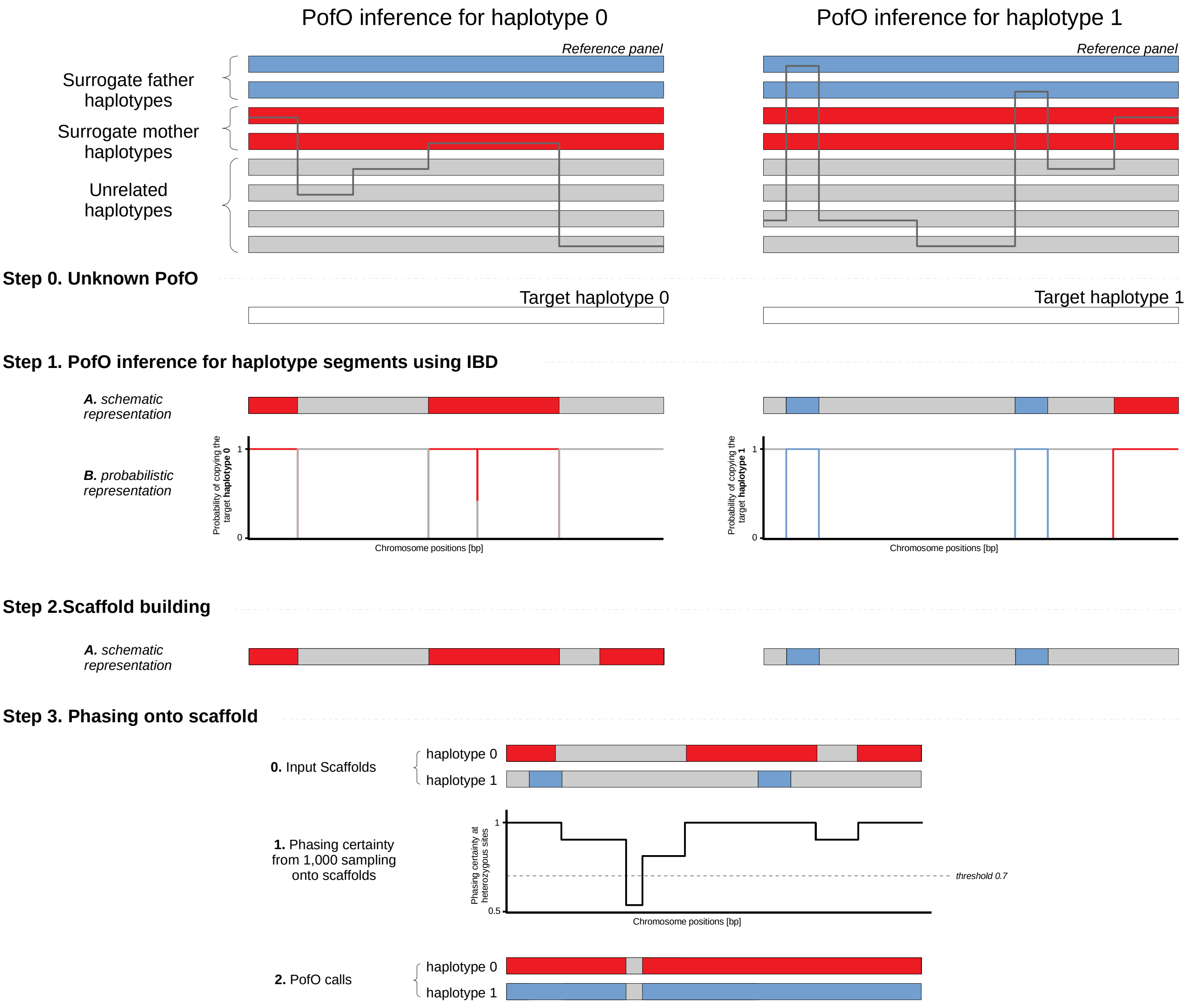

<u>Supplementary Figure 1:</u> PofO inference from surrogate parents.
Schematic representation of the PofO inference performed for each of the two haplotypes of a target sample. In **step 1**, the PofO of haplotype segments is inferred from the surrogate parents using a HMM that computes IBD sharing between the target haplotype and a reference panel comprising haplotypes from the surrogate parents and 100 unrelated haplotypes. In **step 2**, the resulting IBD segments are used to assemble a haplotype scaffold to inform another round of phasing. In **step 3**, we sampled 1,000 haplotype pairs per target as part of the last phasing round in order to compute the frequency at which unassigned alleles co-localize onto the maternal/paternal scaffold, thereby deriving confidence scores for their PofO extrapolation.

a.

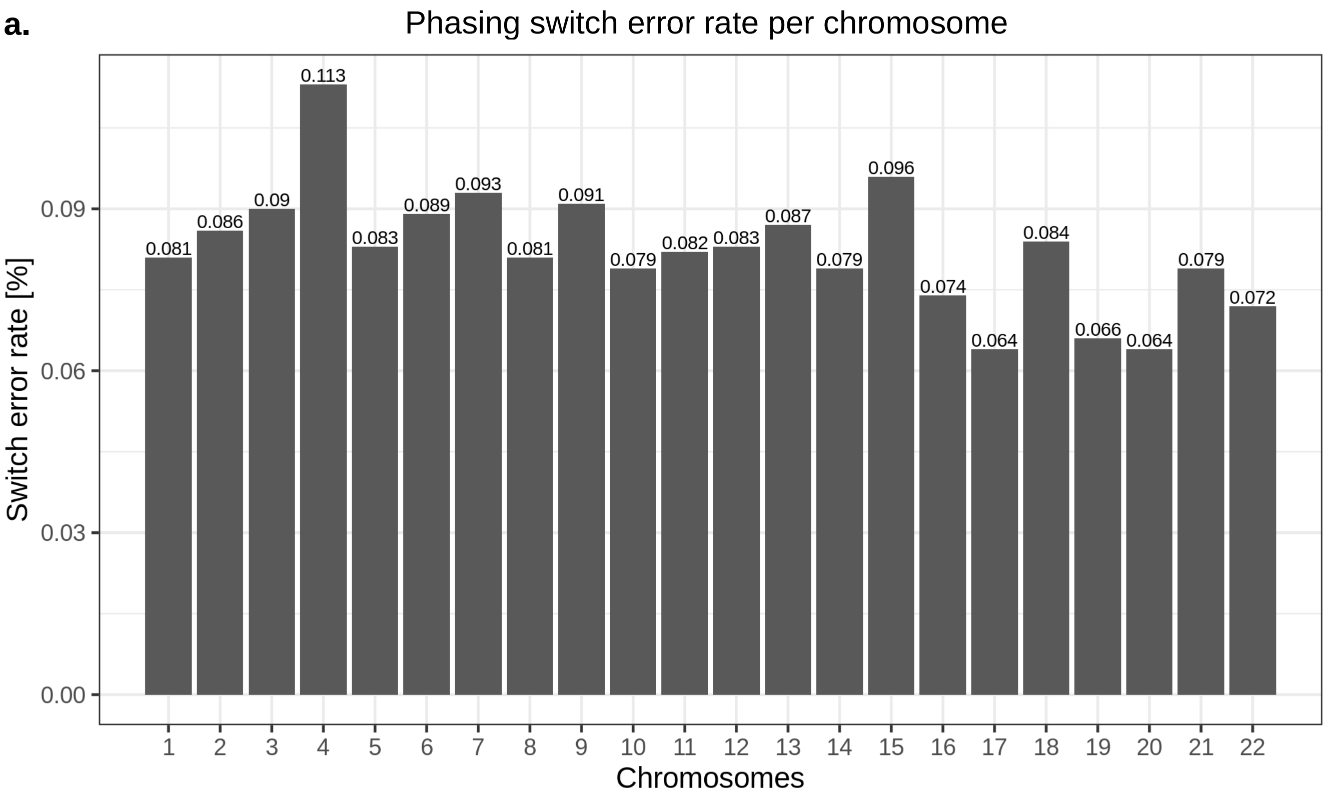

b.

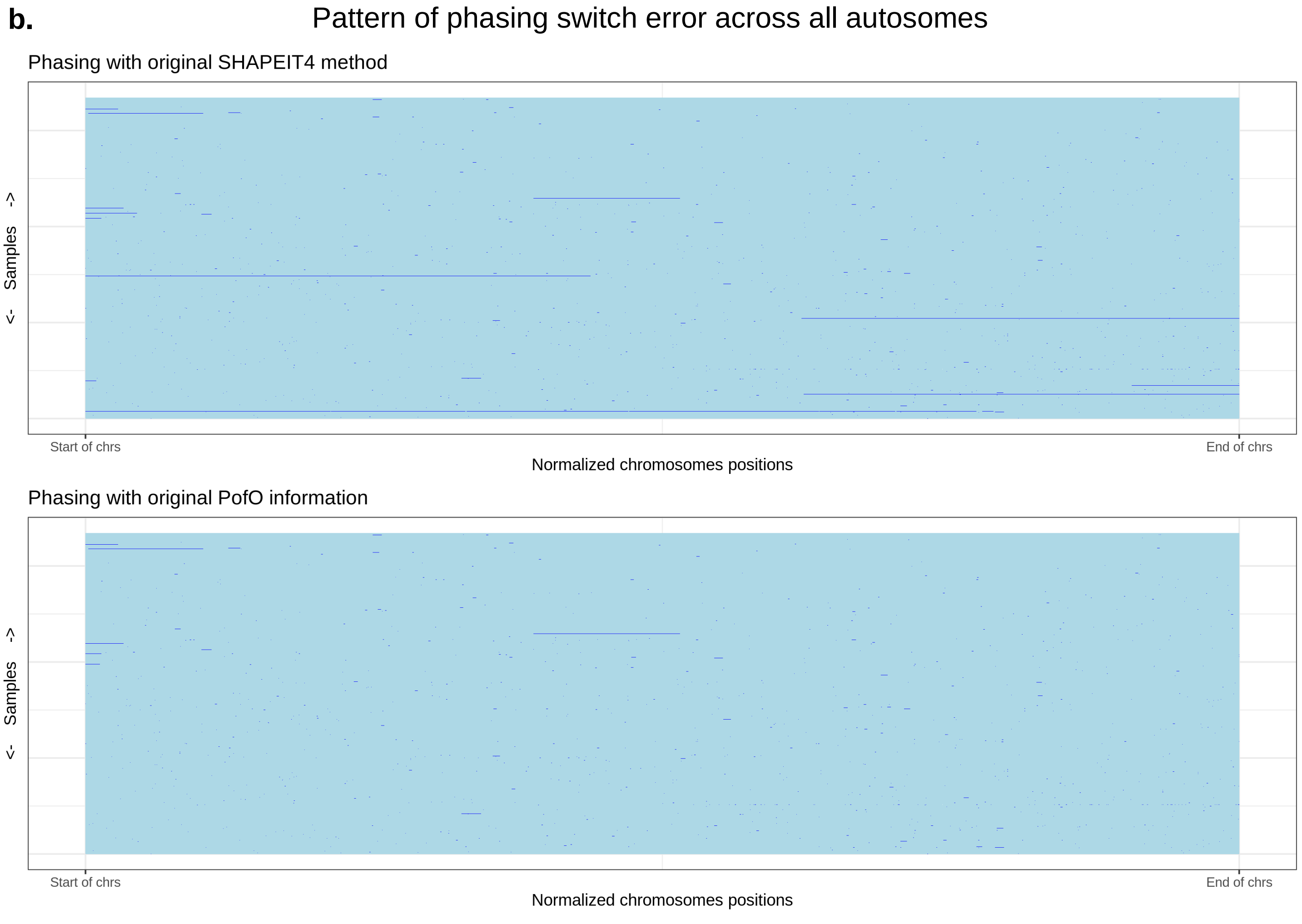

__Supplementary Figure 2:__ **Phasing accuracy with known PofO information.**
a. Phasing switch error rates per chromosome. **b.** Genomic location of switch errors along the chromosomes for 308 samples with both surrogate parents and genotyped parents (y-axis). Positions are normalized between 0 and 1 (x-axis). Continuous segments with the same color represent segments correctly phased. Switches between two colors represent switch errors.

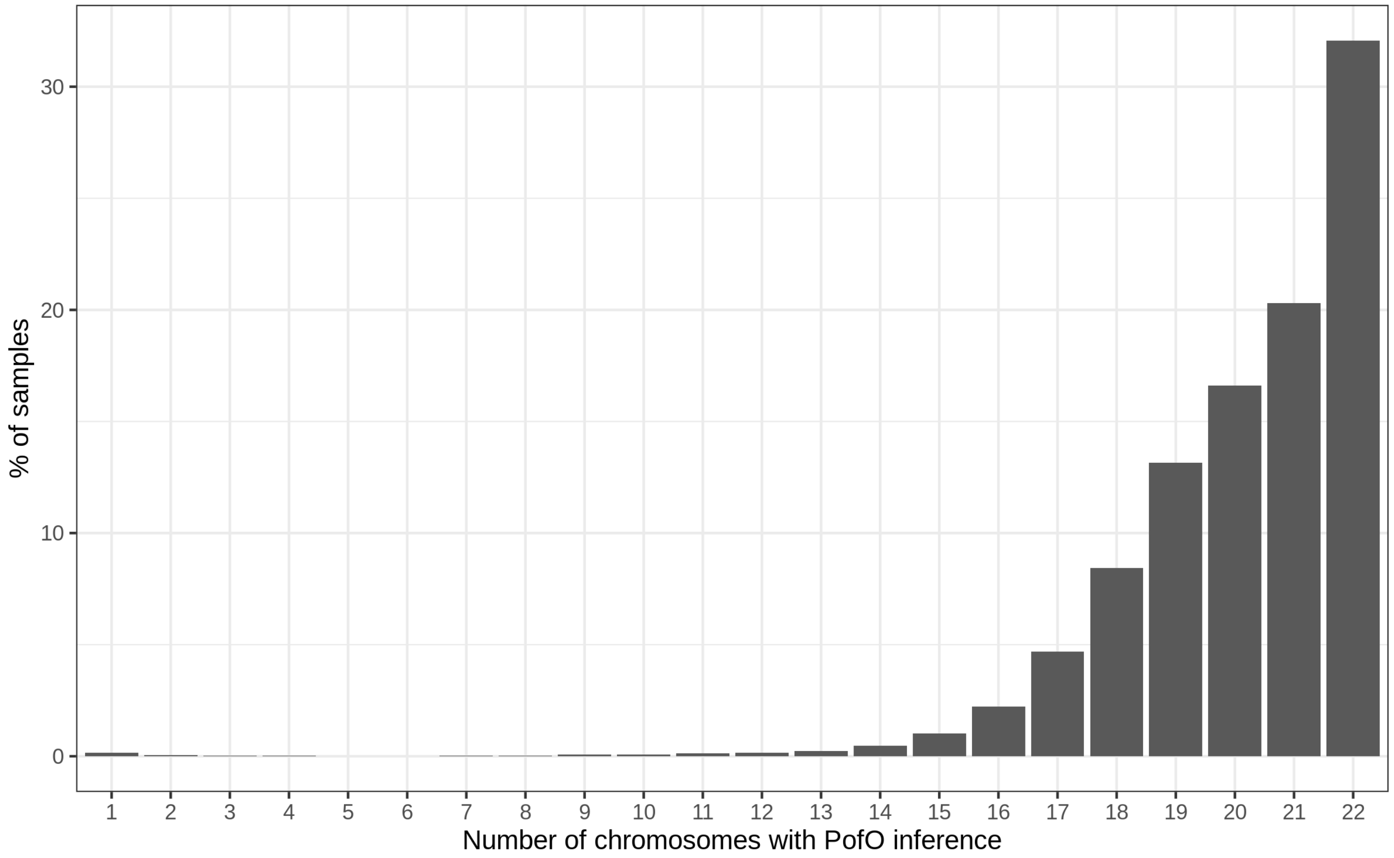

<u>Supplementary Figure 3:</u> Coverage of PofO inference across samples.
Proportion of samples (y-axis) having PofO inference across N chromosomes (x-axis).

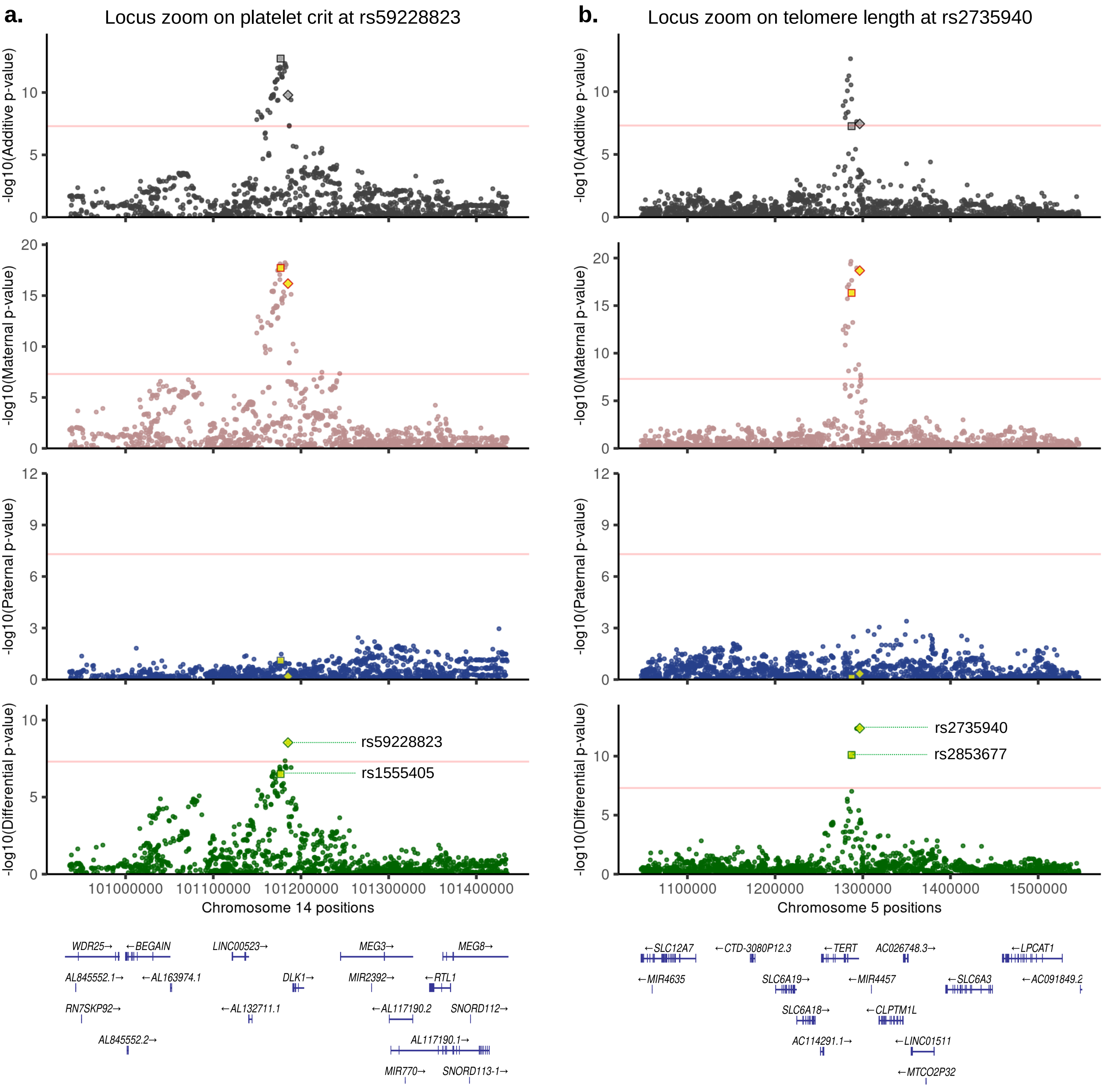

<u>Supplementary Figure 4:</u> Locus zoom

Locus zoom on (**a.**) platelet crit at rs59228823 and (**b.**) telomere length at rs2735940 along a 500kb window centered on the variant (diamond) across the additive (black), maternal (red), paternal (blue) and differential (green) scans. Alternative causal variants previously reported and assessed in this study are shown with a square.

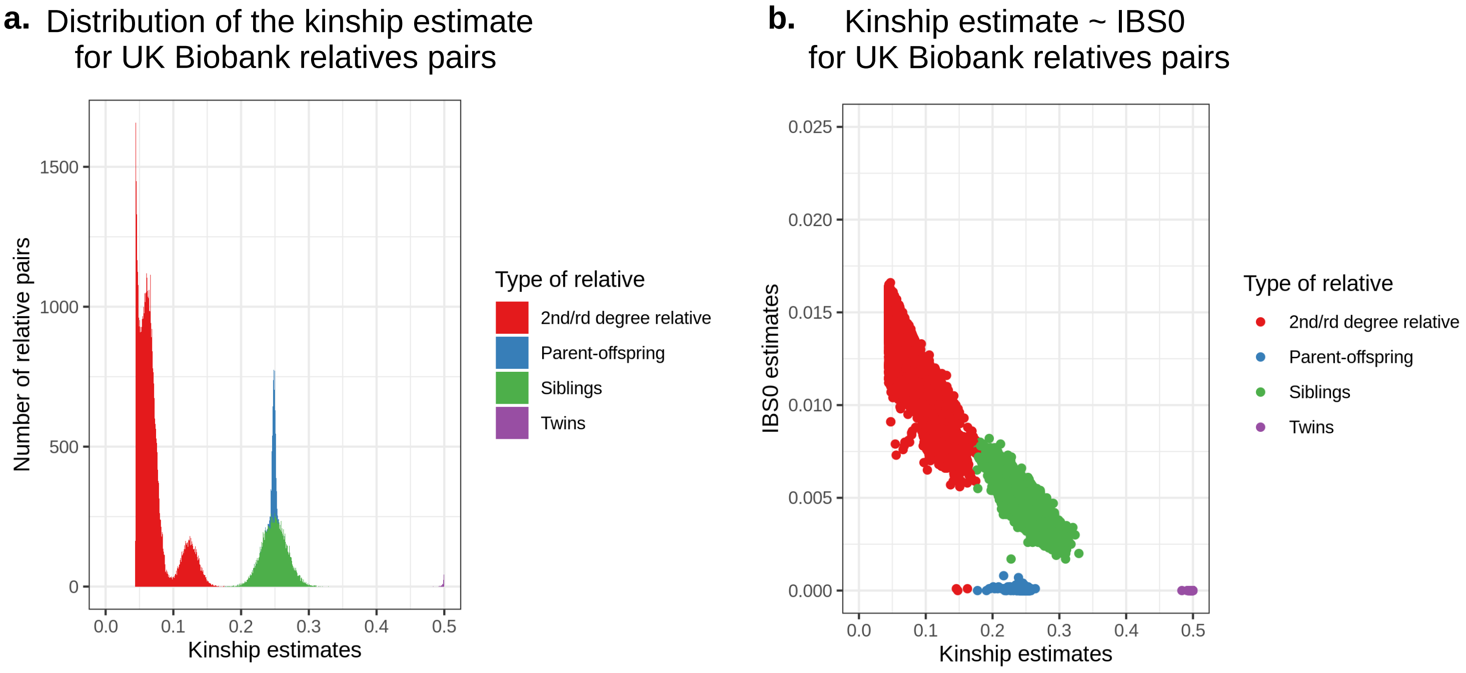

<u>Supplementary Figure 5:</u> Relatedness in UK biobank.
a. Distribution of the kinship estimate across all UK Biobank relative pairs up to the third degree.
b. Kinship estimates in function of the IBS0 estimates in all UK biobank relative pairs up to the third degree. Colors indicate the classification used in our analysis.

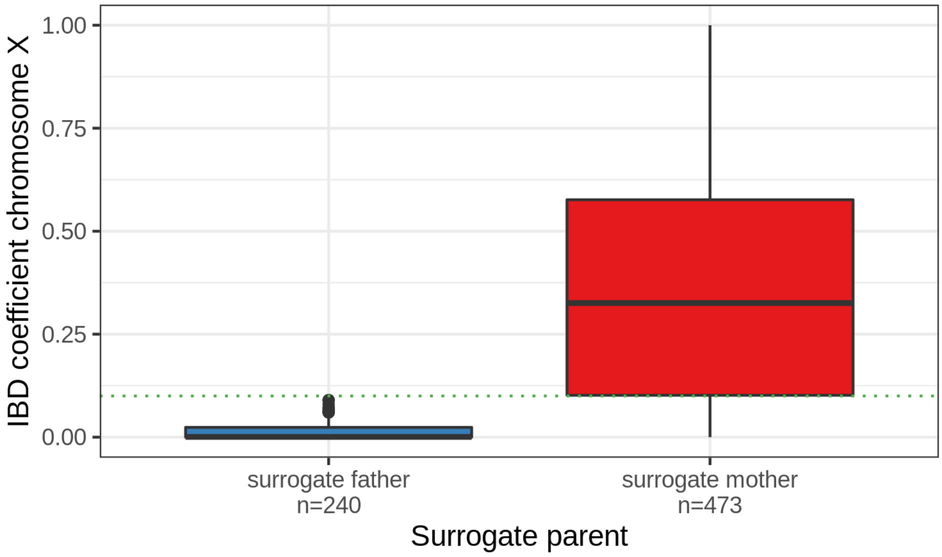

<u>Supplementary Figure 6:</u> Validation of chromosome X IBD.
IBD coefficient on chromosome X (y-axis) for targets with genotyped parents and surrogate parents (i.e. validation cohort). The IBD is estimated between these targets and their surrogate mother or surrogate father (x-axis). Dotted green line indicates the cut-off used in our analysis (=0.1). Boxes bound the 25th, 50th (median) and 75th quantile. Whiskers range from minima (lower) and maxima (upper).

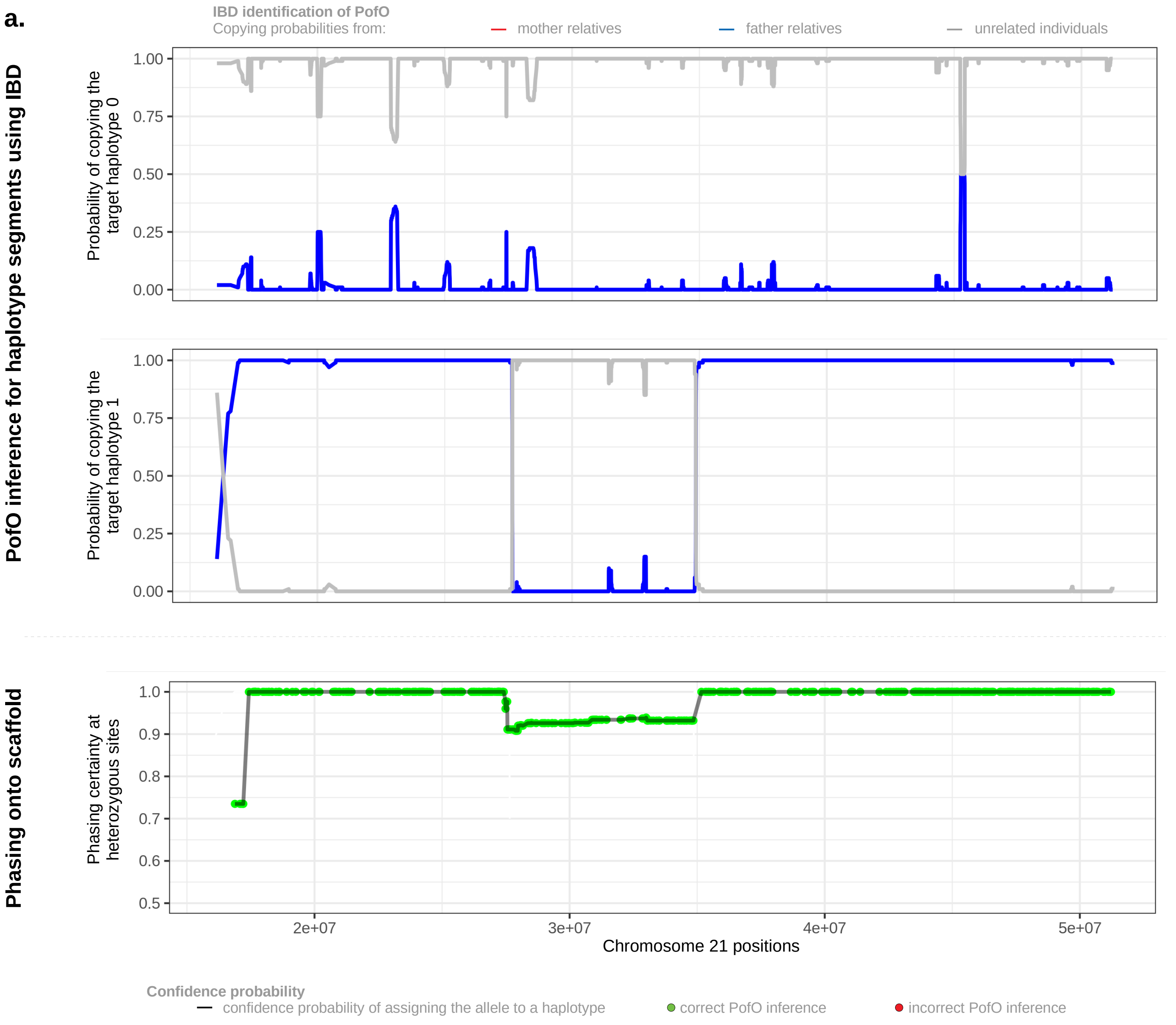
a.
IBD identification of PofO
Copying probabilities from:
mother relatives
father relatives
unrelated individuals
PofO inference for haplotype segments using IBD
Probability of copying the target haplotype 0
Probability of copying the target haplotype 1
1.00
0.75
0.50
0.25
0.00
Phasing onto scaffold
Phasing certainty at heterozygous sites
1.0
0.9
0.8
0.7
0.6
0.5
2e+07
3e+07
4e+07
5e+07
Chromosome 21 positions
Confidence probability
confidence probability of assigning the allele to a haplotype
correct PofO inference
incorrect PofO inference

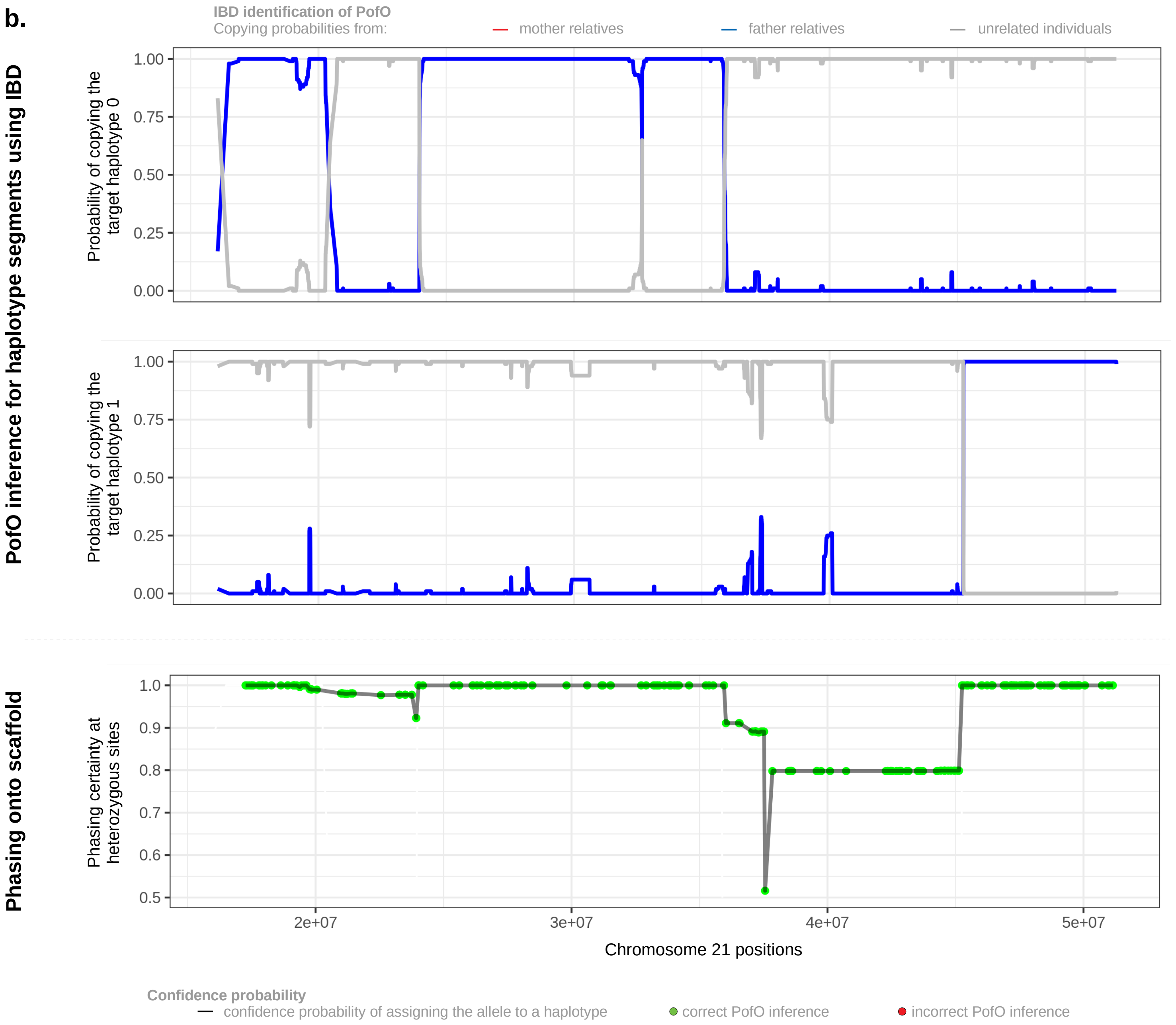

b.
IBD identification of PofO
Copying probabilities from:
mother relatives
father relatives
unrelated individuals
PofO inference for haplotype segments using IBD
Probability of copying the target haplotype 0
Probability of copying the target haplotype 1
1.00
0.75
0.50
0.25
0.00
Phasing onto scaffold
Phasing certainty at heterozygous sites
1.0
0.9
0.8
0.7
0.6
0.5
2e+07
3e+07
4e+07
5e+07
Chromosome 21 positions
Confidence probability
confidence probability of assigning the allele to a haplotype
correct PofO inference
incorrect PofO inference

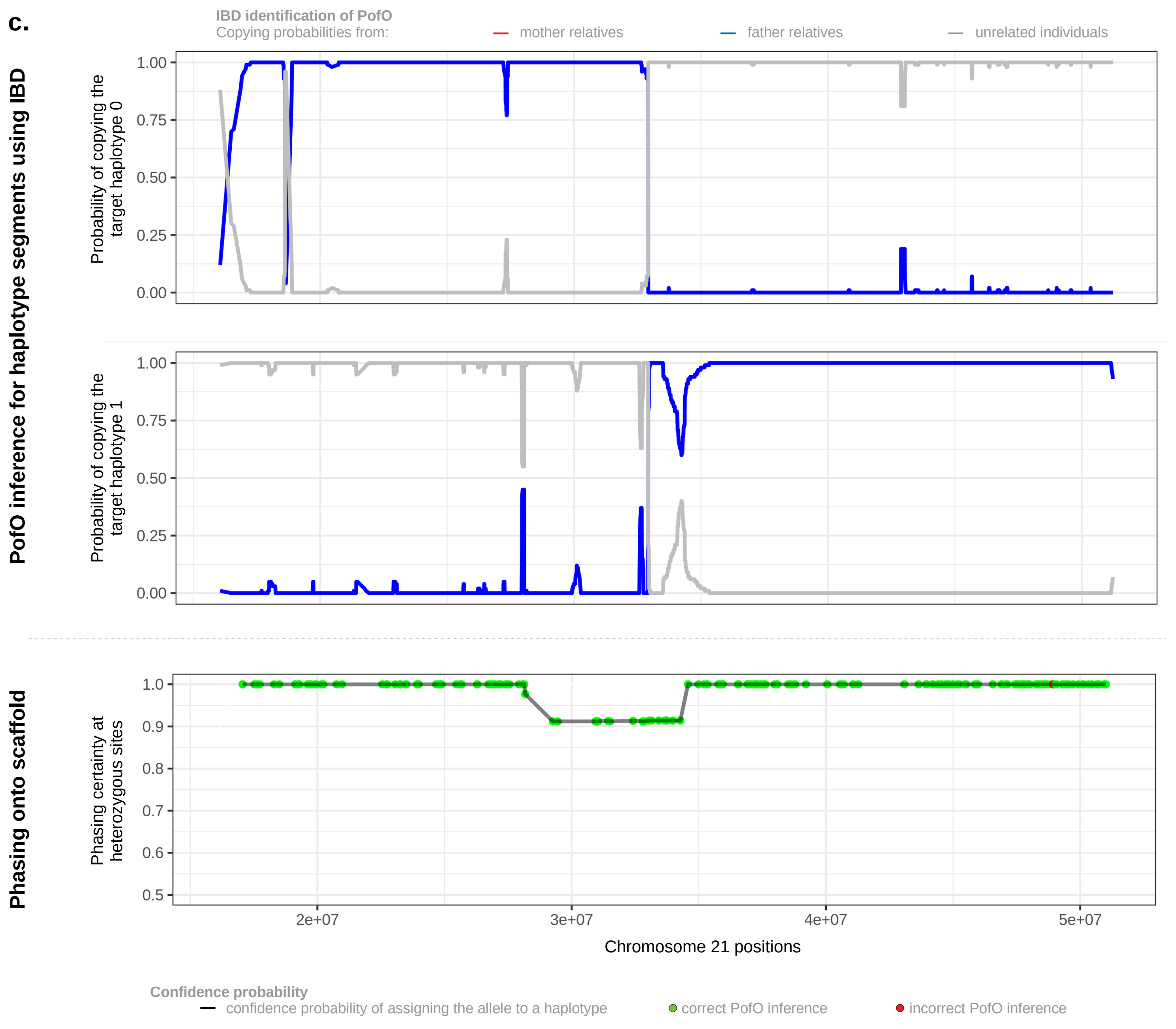

c.
PofO inference for haplotype segments using IBD
IBD identification of PofO
Copying probabilities from:
mother relatives
father relatives
unrelated individuals
Probability of copying the target haplotype 0
1.00
0.75
0.50
0.25
0.00
Probability of copying the target haplotype 1
1.00
0.75
0.50
0.25
0.00
Phasing onto scaffold
Phasing certainty at heterozygous sites
1.0
0.9
0.8
0.7
0.6
0.5
2e+07
3e+07
4e+07
5e+07
Chromosome 21 positions
Confidence probability
confidence probability of assigning the allele to a haplotype
correct PofO inference
incorrect PofO inference

d.

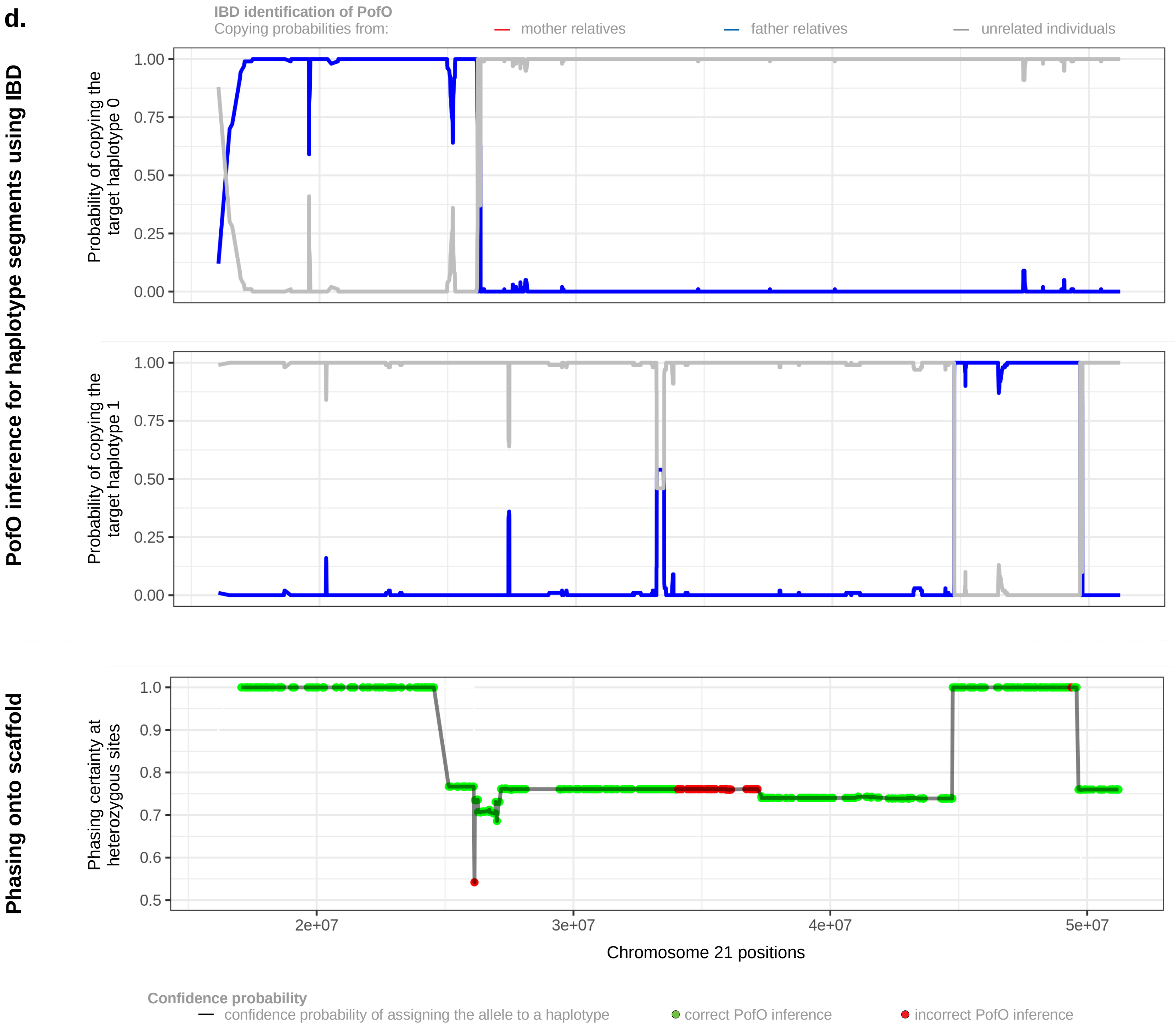

IBD identification of PofO
Copying probabilities from:
mother relatives
father relatives
unrelated individuals
PofO inference for haplotype segments using IBD
Probability of copying the target haplotype 0
Probability of copying the target haplotype 1
1.00
0.75
0.50
0.25
0.00
Phasing onto scaffold
Phasing certainty at heterozygous sites
1.0
0.9
0.8
0.7
0.6
0.5
2e+07
3e+07
4e+07
5e+07
Chromosome 21 positions
Confidence probability
confidence probability of assigning the allele to a haplotype
correct PofO inference
incorrect PofO inference

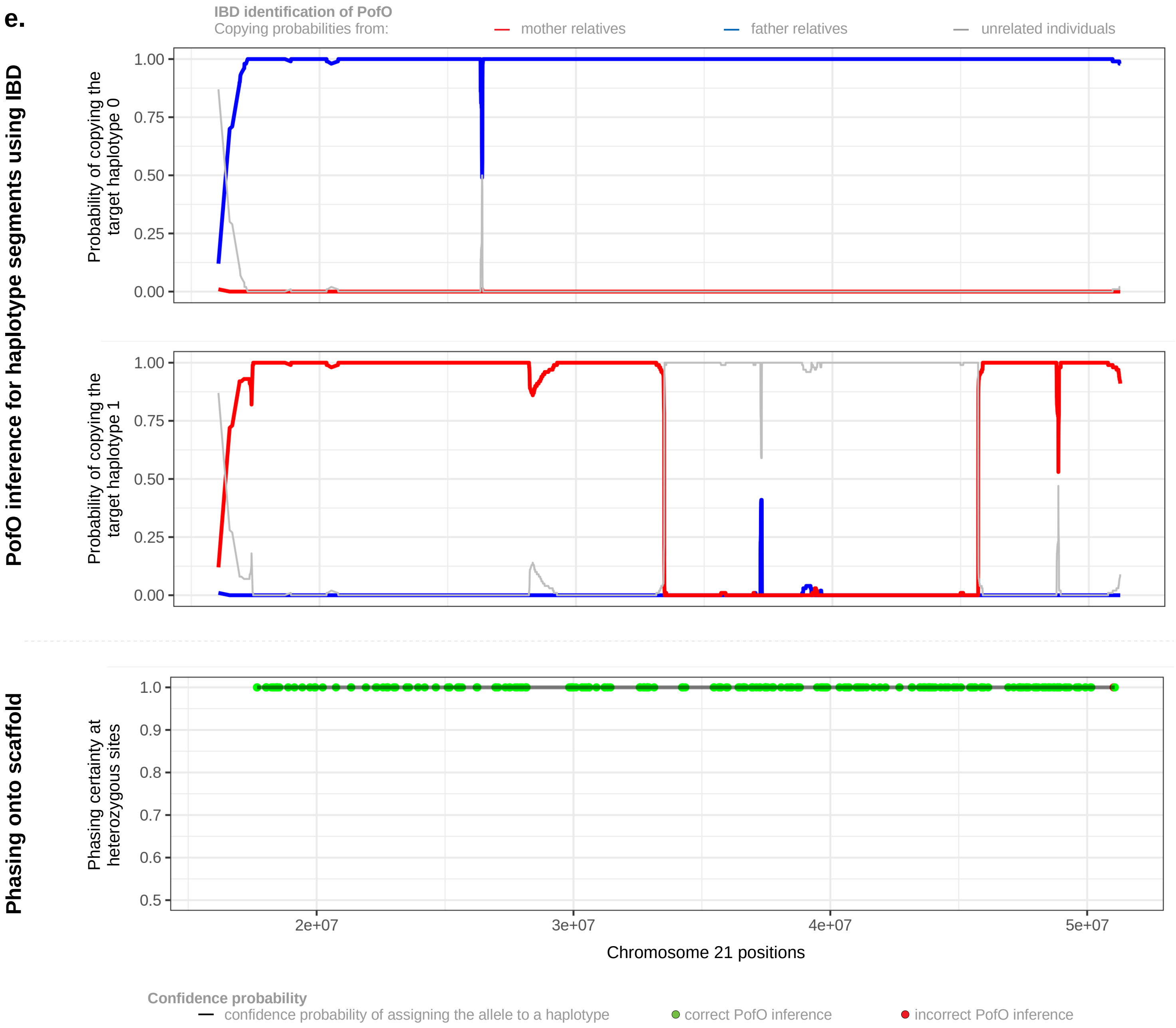
e.
PofO inference for haplotype segments using IBD
IBD identification of PofO
Copying probabilities from:
mother relatives
father relatives
unrelated individuals
Probability of copying the target haplotype 0
Probability of copying the target haplotype 1
1.00
0.75
0.50
0.25
0.00
Phasing onto scaffold
Phasing certainty at heterozygous sites
1.0
0.9
0.8
0.7
0.6
0.5
2e+07
3e+07
4e+07
5e+07
Chromosome 21 positions
Confidence probability
confidence probability of assigning the allele to a haplotype
correct PofO inference
incorrect PofO inference

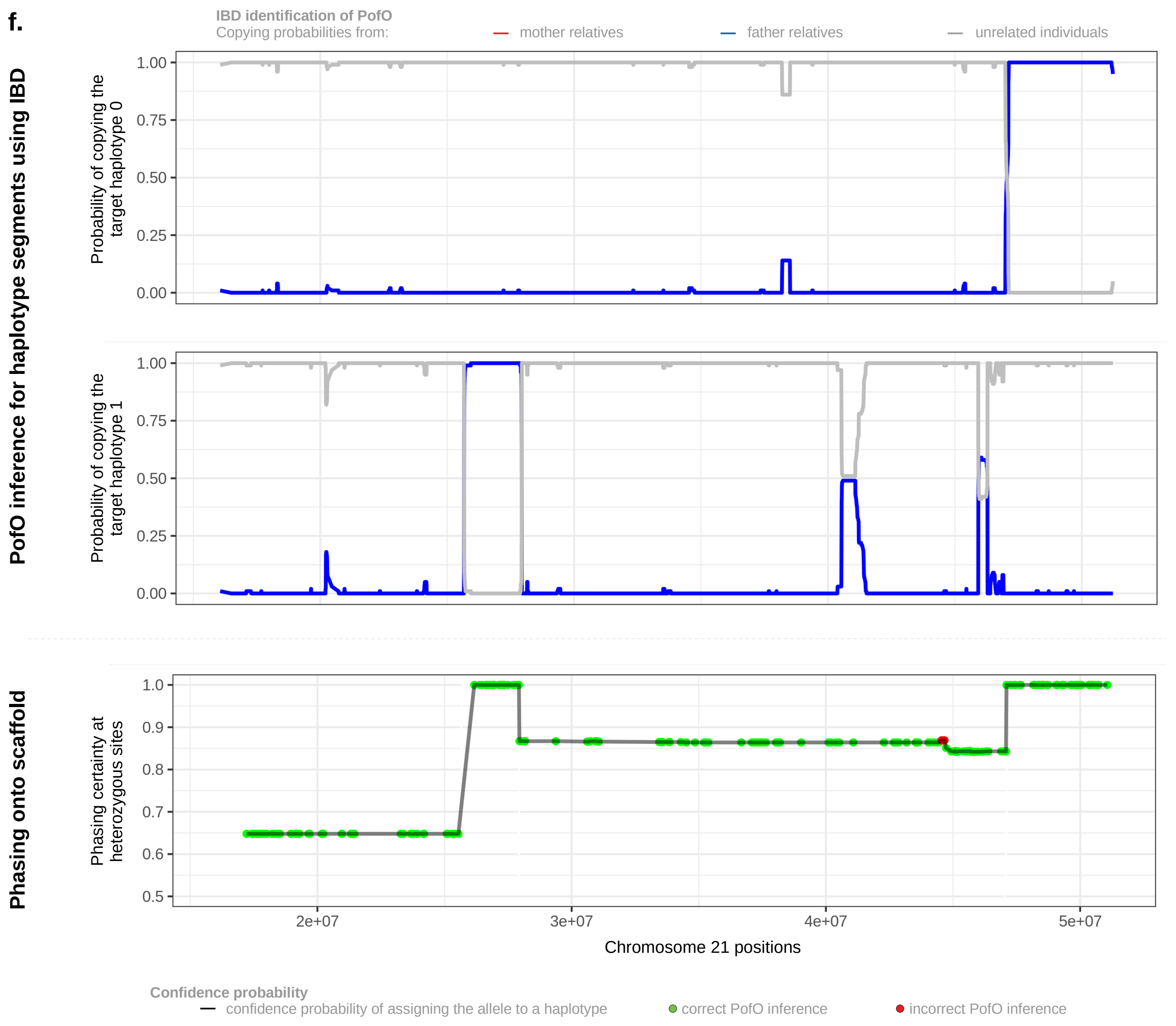

f.
PofO inference for haplotype segments using IBD
IBD identification of PofO
Copying probabilities from:
mother relatives
father relatives
unrelated individuals
Probability of copying the target haplotype 0
Probability of copying the target haplotype 1
1.00
0.75
0.50
0.25
0.00
Phasing onto scaffold
Phasing certainty at heterozygous sites
1.0
0.9
0.8
0.7
0.6
0.5
2e+07
3e+07
4e+07
5e+07
Chromosome 21 positions
Confidence probability
confidence probability of assigning the allele to a haplotype
correct PofO inference
incorrect PofO inference

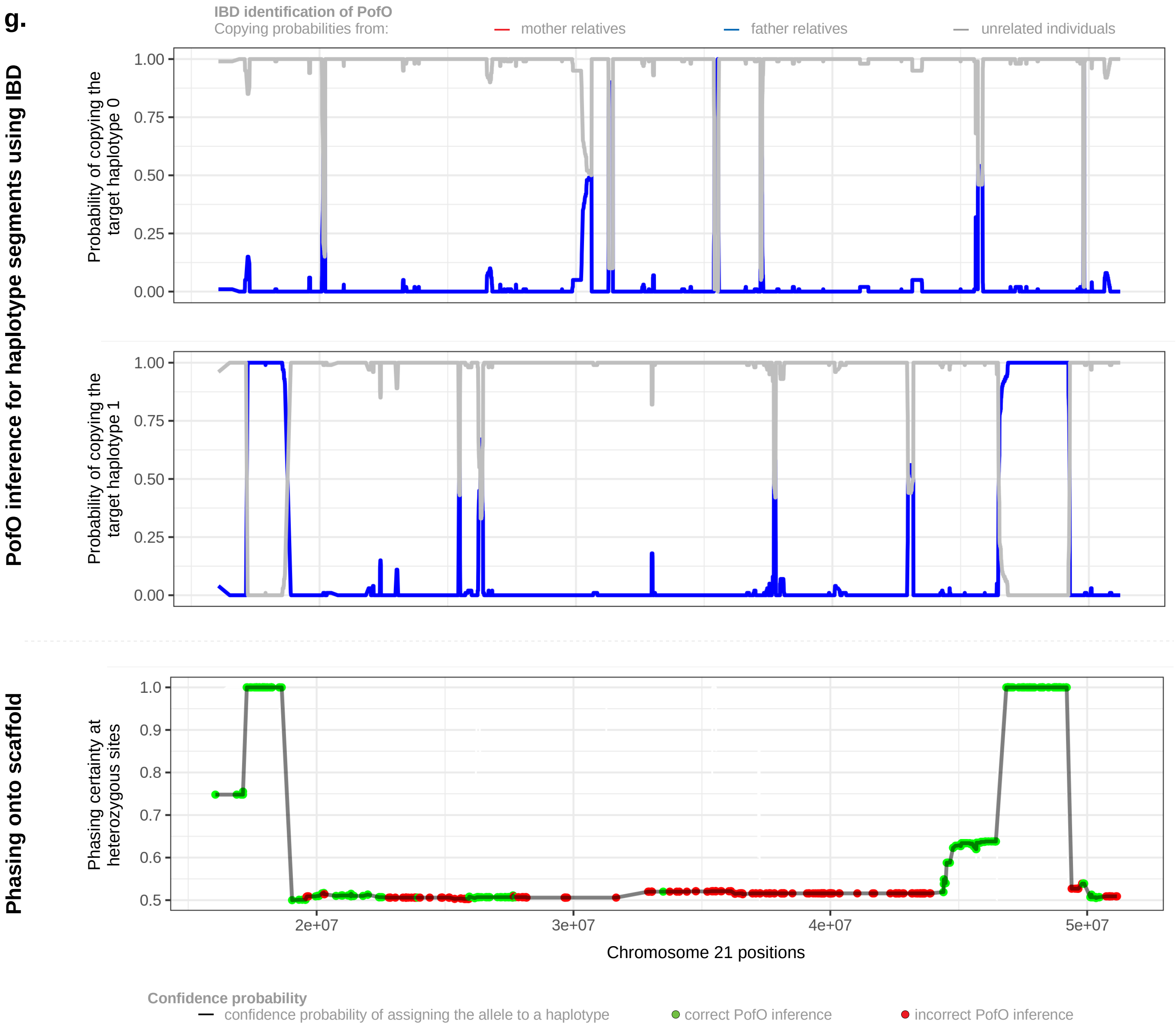

<u>Supplementary Figure 7a-g.</u> PofO inference from the surrogate parents for chromosome 21 across 8 validation individuals. Panels 1-2 show the probability given by our HMM (y-axis) that each target haplotype copies from the surrogate father(s) in blue, the surrogate mother(s) in red or unrelated individuals in grey as we move along chromosome 21. Panel 3 shows the phasing certainty resulting from 1,000 sampling onto scaffold. Red dots show errors in the PofO inference while green dots show correct PofO inference

Supplementary tables

	Number of individuals	Number of individuals with PofO inferred from both parental genome (i.e trios)	Number of individuals with PofO inferred from one parental genome (i.e duos)	Number of individuals with PofO inferred from surrogate parents and chromosome X mapping	Number of individuals with PofO deduced from siblings	mean age	sd age	median age	min age	max age
Both sex	26393	1037	3872	20620	864	54.7	9.13	56	39	73
Males	22652	427	1516	20620	89	56.25	8.62	58	39	73
Females	3741	610	2356	0	775	46.05	6.81	44	39	70

Supplementary Table 1. Study samples

Phenotype	SNP	Chr	Position (hg19)	Risk allele	MAF	Add.P	Add.B	Pat.P	Pat.B	Mat.P	Mat.B	Diff.P	Mapped gene	UKB phenotype code	Hoggart *et al.*, p-values	PofO effect
Mean platelet volume	rs17388591	2	7611948	A	0.1511	0.11	0.0151	2.8e-06	0.0755	0.0034	-0.0486	3.3e-08	intergenic	30100	0.0020062	paternal
Platelet crit	rs116403284	2	21812980	T	0.0325	0.05	-0.0445	1.3e-05	-0.158	0.23	0.0456	3.4e-08	intergenic	30090	0.00365278	paternal
Sitting height	rs934235	2	105661013	G	0.4639	0.31	0.0063	1.5e-06	0.046	0.001	-0.0320	2.9e-09	MRPS9	20015	0.00172474	paternal
Eosinophil percentage	rs77403171	2	143984854	G	0.0102	0.64	0.0218	0.00085	0.229	0.0002	-0.256	1.6e-08	ARHGAP15	30210	0.00634692	opposite
Leg fat-free mass (left)	rs117968021	8	79223827	T	0.0117	0.85	0.0062	8.6e-05	0.151	0.00061	-0.128	4.5e-08	PKIA-AS1	23117	0.00585272	opposite
Mean corpuscular volume	rs10763958	10	34170183	C	0.4968	0.51	0.0091	1.0e-05	0.0608	0.0005	-0.0418	8.8e-09	intergenic	30040	0.00174499	opposite
Sitting height	rs143543103	10	70040971	T	0.0432	0.24	0.0233	0.0029	-0.0680	8.7e-06	0.116	5.5e-09	PBLD, MYPN	20015	0.00321167	maternal
Platelet crit	rs1790192	11	118757191	G	0.3933	0.56	-0.0065	0.0098	0.0312	0.00063	-0.0452	3.9e-08	CXCR5	30090	0.00175238	opposite
Total bilirubin	rs62032857	16	20338450	A	0.0247	0.52	0.0317	0.0022	-0.095	2.2e-06	0.193	3.7e-08	GP2	30840	0.00405805	maternal
Direct bilirubin	rs62032857	16	20338450	A	0.0247	0.83	0.0071	8.3e-05	-0.147	8.2e-05	0.175	2.6e-08	GP2	30660	0.00437831	opposite
Impedance of arm (left)	rs432573	19	16304742	T	0.1527	0.56	-0.0051	1.1e-05	-0.058	0.00096	0.0447	4.2e-08	FAM32A, AP1M1, enhancer	23110	0.00207257	opposite
Impedance of arm (right)	rs432573	19	16304742	T	0.1527	0.71	-0.0029	1.7e-05	-0.056	0.00067	0.0458	4,00E-08	FAM32A, AP1M1, enhancer	23109	0.00207230	opposite
Nucleated red blood cell percentage	rs12983350	19	31383551	A	0.0272	0.00043	0.0284	5.7e-10	0.0754	0.66	-0.0052	4.4e-09	intergenic	30230	0.00658008	paternal
Nucleated red blood cell count	rs12985289	19	31383552	A	0.0272	0.001	0.0265	7.6e-10	0.0750	0.45	-0.0091	6.5e-09	intergenic	30170	0.00658255	paternal

Supplementary Table 2. Putative PofO associations not supported by additive associations. P-values are computed using BOLT-MM[1]. Add=Additive ; Pat.=Paternal ; Mat.=Maternal ; Diff.=Differential ; P=p-values; B=betas ; Chr=Chromosome ; MAF=minor allele frequency.

SNP	Chr	Position (hg19)	Risk allele	MAF	Add.P	Add.B	Pat.P	Pat.B	Mat.P	Mat.B	Diff.P	Phenotype	UKB phenotype code	Pofo effect
rs527065	1	177868882	T	0.3286	1.3e-07	0.0409	1.8e-09	0.067	0.11	0.018	0.0015	Weight	23098	paternal
rs539515	1	177889025	C	0.208	5.3e-08	0.0539	2.6e-09	0.086	0.057	0.027	0.0043	Arm fat mass (right)	23120	paternal
rs539515	1	177889025	C	0.208	1.3e-06	0.036	4.6e-08	0.060	0.16	0.016	0.0044	Arm fat percentage (right)	23119	paternal
rs8030	1	177897975	T	0.3035	2.2e-07	0.0401	7e-09	0.065	0.091	0.018	0.0032	Waist circumference	48	paternal
rs8030	1	177897975	T	0.3035	7.5e-07	0.0392	1.8e-08	0.06	0.12	0.018	0.0035	Leg fat mass (left)	23116	paternal
rs8030	1	177897975	T	0.3035	2.7e-07	0.041	1.2e-08	0.066	0.077	0.021	0.0047	Leg fat mass (right)	23112	paternal
rs531385	1	177904428	C	0.3502	1e-06	0.0404	4.8e-09	0.071	0.2	0.014	0.00078	Hip circumference	49	paternal
rs531385	1	177904428	C	0.3502	5.8e-08	0.0411	8.2e-10	0.067	0.083	0.019	0.0011	Weight	21002	paternal
rs531385	1	177904428	C	0.3502	5e-07	0.0280	1.2e-08	0.046	0.09	0.014	0.0029	Arm fat-free mass (left)	23125	paternal
rs531385	1	177904428	C	0.3502	6.8e-07	0.028	1.8e-08	0.046	0.086	0.0141	0.0039	Arm predicted mass (left)	23126	paternal
rs531385	1	177904428	C	0.3502	6.7e-07	0.0283	3.7e-08	0.045	0.088	0.0146	0.0049	Basal metabolic rate	23105	paternal

Supplementary Table 3. PheWAS at 1q25.2. P-values are computed using BOLT-MM[1]. Add=Additive ; Pat.=Paternal ; Mat.=Maternal ; Diff.=Differential ; P=p-values; B=betas; Chr=Chromosome ; MAF=minor allele frequency.

Supplementary References

1. Loh, P.R. *et al.* Efficient Bayesian mixed-model analysis increases association power in large cohorts. *Nat Genet* **47**, 284-90 (2015).
