# Supplementary material for: Parent-of-Origin inference for biobanks
Source: Nat Commun. 2022 Nov 5;13:6668. doi: 10.1038/s41467-022-34383-6 (PMC9637181; doi:10.1038/s41467-022-34383-6)
Supplement: Supplementary file 3 — Description of Additional Supplementary Files [file 41467_2022_34383_MOESM3_ESM.pdf]

Description of Additional Supplementary Files

Supplementary Data submitted in one Excel file called "Supplementary Data 1-3" with 3 tabs:

Supplementary Data 1. Distribution of the phenotypes tested in this study across all individuals, or stratified by sex. N=number of samples; Mean=mean; sd= standard deviation; min=minimum; max=maximum.

Supplementary Data 2. Phenome-wide association study across the four reported type-2-diabetes variants. P-values (in our study) are computed using BOLT-MM[18]. Add=Additive ; Pat.=Paternal ; Mat.=Maternal ; Diff.=Differential ; P=p-values; B=betas.

Supplementary Data 3. Replication of PofO associations with birth weight reported by Juliusdottir et al[41] . P-values (in our study) are computed using BOLT-MM[18]. Add=Additive ; Pat.=Paternal ; Mat.=Maternal ; Diff.=Differential ; P=p-values; B=betas.
