# Supplementary material for: Parent-of-Origin inference for biobanks
Source: Nat Commun. 2022 Nov 5;13:6668. doi: 10.1038/s41467-022-34383-6 (PMC9637181; doi:10.1038/s41467-022-34383-6)
Supplement: Supplementary file 5 — Reporting Summary [file 41467_2022_34383_MOESM5_ESM.pdf]

nature portfolio

Corresponding author(s): Olivier Delaneau

Last updated by author(s): Sep 20, 2022

Reporting Summary

Nature Portfolio wishes to improve the reproducibility of the work that we publish. This form provides structure for consistency and transparency in reporting. For further information on Nature Portfolio policies, see our Editorial Policies and the Editorial Policy Checklist.

Statistics

For all statistical analyses, confirm that the following items are present in the figure legend, table legend, main text, or Methods section.

n/a	Confirmed	
☐	☒	The exact sample size (*n*) for each experimental group/condition, given as a discrete number and unit of measurement
☒	☐	A statement on whether measurements were taken from distinct samples or whether the same sample was measured repeatedly
☐	☒	The statistical test(s) used AND whether they are one- or two-sided<br>*Only common tests should be described solely by name; describe more complex techniques in the Methods section.*
☐	☒	A description of all covariates tested
☐	☒	A description of any assumptions or corrections, such as tests of normality and adjustment for multiple comparisons
☐	☒	A full description of the statistical parameters including central tendency (e.g. means) or other basic estimates (e.g. regression coefficient) AND variation (e.g. standard deviation) or associated estimates of uncertainty (e.g. confidence intervals)
☐	☒	For null hypothesis testing, the test statistic (e.g. *F*, *t*, *r*) with confidence intervals, effect sizes, degrees of freedom and *P* value noted<br>*Give P values as exact values whenever suitable.*
☐	☒	For Bayesian analysis, information on the choice of priors and Markov chain Monte Carlo settings
☒	☐	For hierarchical and complex designs, identification of the appropriate level for tests and full reporting of outcomes
☒	☐	Estimates of effect sizes (e.g. Cohen's *d*, Pearson's *r*), indicating how they were calculated

Our web collection on statistics for biologists contains articles on many of the points above.

Software and code

Policy information about availability of computer code

Data collection	No software was used for the data collection of this study.
Data analysis	Publicly available code used in this study :<br>BCFtools v1.8 (based on HTSlib v1.8)<br>SHAPEIT v4.2.1 (https://github.com/odelaneau/shapeit4)<br>XIBD v1.0.0 (https://github.com/bahlolab/XIBD)<br>BOLT-LMM v2.3.4 (https://alkesgroup.broadinstitute.org/BOLT-LMM/downloads/)<br>PLINK v1.90b5<br>R v3.5.1<br>R 'igraph' package v1.2.2<br>QUICKTEST v1.1<br>Parent-of-Origin HMM designed for this study (https://github.com/RJHFMSTR/PofO_inference/tree/master/HMM)<br><br>Non publicly available code used in this study:<br>IMPUTE5 v1.1.4

For manuscripts utilizing custom algorithms or software that are central to the research but not yet described in published literature, software must be made available to editors and reviewers. We strongly encourage code deposition in a community repository (e.g. GitHub). See the Nature Portfolio guidelines for submitting code & software for further information.

Data

Policy information about availability of data

All manuscripts must include a data availability statement. This statement should provide the following information, where applicable:

- Accession codes, unique identifiers, or web links for publicly available datasets
- A description of any restrictions on data availability
- For clinical datasets or third party data, please ensure that the statement adheres to our policy

The publicly available subset of the Haplotype Reference Consortium dataset is available from the European Genome-Phenome Archive at the European Bioinformatics Institute, accession EGAS00001001710.

The UK Biobank data was accessed under the project 66995.

We used additional publicly available databases that have been consulted multiple time between September 2021 and March 2022:
- GeneImprint (http://www.geneimprint.com/)
- Catalog of Imprinted Genes (http://www.otago.ac.nz/IGC)
- UK Biobank phenotype correlations (https://ukbb-rg.hail.is/)

Field-specific reporting

Please select the one below that is the best fit for your research. If you are not sure, read the appropriate sections before making your selection.

☒ Life sciences ☐ Behavioural & social sciences ☐ Ecological, evolutionary & environmental sciences

For a reference copy of the document with all sections, see nature.com/documents/nr-reporting-summary-flat.pdf

Life sciences study design

All studies must disclose on these points even when the disclosure is negative.

Sample size	The sample size of our study is 26,393 individuals, that is, the number of individuals for which we infer the parent-of-origin using the method desccribed in the manuscript. The sample sizes of the association tests (GWAS) varie from that as not all the 26,393 individuals have all the 99 phenotype measurements available. The GWAS sample size for each phenotypes is provided in supplementary data 1.
Data exclusions	Initially we filtered out all variants that were not included for the phasing of the original UK Biobank release. These were determined from the SNPs QC file provided as part of the UK Biobank, ressource 1955.
Replication	Most of the softwares used for this study are publicly available.
Randomization	Randomization was not used since there are no experimental groups.
Blinding	Blinding is not relevant to this study since no group allocation occurs.

Reporting for specific materials, systems and methods

We require information from authors about some types of materials, experimental systems and methods used in many studies. Here, indicate whether each material, system or method listed is relevant to your study. If you are not sure if a list item applies to your research, read the appropriate section before selecting a response.

Materials & experimental systems

n/a	Involved in the study	
☒	☐	Antibodies
☒	☐	Eukaryotic cell lines
☒	☐	Palaeontology and archaeology
☒	☐	Animals and other organisms
☐	☒	Human research participants
☒	☐	Clinical data
☒	☐	Dual use research of concern

Methods

n/a	Involved in the study	
☒	☐	ChIP-seq
☒	☐	Flow cytometry
☒	☐	MRI-based neuroimaging

Human research participants

Policy information about studies involving human research participants

Population characteristics	We used UK Biobank individuals of British or Irish ancestry only.
Recruitment	The participants were recruited as part of the UK Biobank study.
Ethics oversight	UK Biobank Limited

Note that full information on the approval of the study protocol must also be provided in the manuscript.
